# Transcriptomic Analysis of mRNAs in Human Monocytic Cells Expressing the HIV-1 Nef Protein and Their Exosomes

**DOI:** 10.1155/2015/492395

**Published:** 2015-04-15

**Authors:** Madeeha Aqil, Saurav Mallik, Sanghamitra Bandyopadhyay, Ujjwal Maulik, Shahid Jameel

**Affiliations:** ^1^Virology Group, International Centre for Genetic Engineering and Biotechnology, New Delhi 110067, India; ^2^Department of Oral Medicine and Diagnostic Sciences, College of Dentistry, University of Illinois at Chicago, Chicago, IL 60612, USA; ^3^Machine Intelligence Unit, Indian Statistical Institute, Kolkata 700108, India; ^4^Department of Computer Science and Engineering, Jadavpur University, Kolkata, West Bengal 700032, India; ^5^The Wellcome Trust/DBT India Alliance, Plot No. 19, 8-2-684/3K/19, Road No. 12, Banjara Hills, Hyderabad 500034, India

## Abstract

The Nef protein of human immunodeficiency virus (HIV) promotes viral replication and progression to AIDS. Besides its well-studied effects on intracellular signaling, Nef also functions through its secretion in exosomes, which are nanovesicles containing proteins, microRNAs, and mRNAs and are important for intercellular communication. Nef expression enhances exosome secretion and these exosomes can enter uninfected CD4 T cells leading to apoptotic death. We have recently reported the first miRNome analysis of exosomes secreted from Nef-expressing U937monocytic cells. Here we show genome-wide transcriptome analysis of Nef-expressing U937 cells and their exosomes. We identified four key mRNAs preferentially retained in Nef-expressing cells; these code for MECP2, HMOX1, AARSD1, and ATF2 and are important for chromatin modification and gene expression. Interestingly, their target miRNAs are exported out in exosomes. We also identified three key mRNAs selectively secreted in exosomes from Nef-expressing U937 cells and their corresponding miRNAs being preferentially retained in cells. These are AATK, SLC27A1, and CDKAL and are important in apoptosis and fatty acid transport. Thus, our study identifies selectively expressed mRNAs in Nef-expressing U937 cells and their exosomes and supports a new mode on intercellular regulation by the HIV-1 Nef protein.

## 1. Introduction

The human immunodeficiency virus expresses the prototypic retroviral Gag (capsid), Pol (polymerase), and Env (envelop) proteins. Additionally, it also expresses two regulatory (Rev, Tat) and four accessory (Nef, Vif, Vpr, and Vpu/Vpx) proteins [1]. Of these, Nef is a 27–34 kDa myristoylated protein that is abundantly expressed in the early phase of viral replication cycle. It is a multifunctional protein and a major determinant of disease progression [2]. The Nef protein is primarily localized to cellular membranes, which include the inner surface of plasma membrane, endosomal membranes, and the perinuclear region. This facilitates its interaction with several kinases and adaptor proteins in the endocytic machinery, leading to the modulation of several signaling cascades in infected cells [3]. Nef is also secreted out of cells in vesicles called exosomes [4]. It increases the formation of multivesicular bodies (MVBs), which are sites for exosome biogenesis, thus promoting its own export [5, 6]. We have previously shown that Nef also interacts with the microRNA (miRNA) biogenesis factor Argonaute 2 (Ago2) and interferes with miRNA-mediated gene silencing [7].

Exosomes are 30–100 nm vesicles that are formed by the inward invagination of MVB membranes and are released in the extracellular medium when MVBs fuse with the plasma membrane [8]. These vesicles carry a cargo that includes various mRNAs, miRNAs, and proteins, which vary depending upon the cell of origin [9]. Other cells take up these vesicles, and the exosomal cargo has been shown to affect the physiology of recipient cells [10].

The first report indicating exosomal Nef secretion showed vesicles secreted from HIV infected cells by confocal laser-scanning microscopy and by their sedimentation behaviour [11]. Later, it was found in exosomes from Nef-GFP transfected HEK293 cells, transfected human T cell lines including Jurkat and SupT1 cells, and HIV infected primary as well as transformed cells [5, 12, 13]. We recently showed the presence of Nef-EYFP in exosomes secreted from U937 human monocytic cells that stably expressed this fusion protein [4]. Jurkat T cells can take up Nef-containing exosomes, and the protein was found mainly as punctate cytoplasmic structures [11]. Nef exosomes are likely to enter cells via endocytosis and can also fuse with Nef-deficient HIV-1 virions to restore their infectivity [14]. We demonstrated Nef exosomes to be enriched for miRNAs that target key pathways such as cytokine-cytokine receptor interaction and Jak-STAT and MAPK signaling, as well as those that target the viral genome [4]. Here we report the transcriptomic profile of Nef-EYFP expressing U937 cells and their exosomes and we have used various bioinformatics and statistical tools to identify significantly deregulated mRNAs. Our results show four mRNAs to be preferentially retained in Nef-expressing U937 cells: MECP2 (methyl CpG binding protein 2), HMOX1 (heme oxygenase 1), AARSD1 (alanyl-tRNA synthetase domain-containing protein 1), and ATF2 (activating transcription factor 2). These are important for transcriptional regulation and chromatin modifications (and thus viral latency). We also found apoptosis associated tyrosine kinase and fatty acid transporter mRNAs to be selectively secreted in exosomes from Nef-expressing monocytes. These have the potential to modify the physiology and outcome of recipient cells.

## 2. Materials and Methods

### 2.1. Generation of Retroviruses and Stable Cell Lines

This has been described in detail elsewhere [7]. Briefly, the pEYFP-N1 and pEYFP-Nef-F2 plasmids [15] were digested with BamHI and HpaI, and the released fragments containing the* eyfp* and* nef-eyfp* genes, respectively, were cloned into BglII and HpaI sites in the pMSCV retroviral transfer plasmid. The positive clones were confirmed by restriction digestion and analyzed for EYFP or Nef-EYFP expression by transient transfection in HEK293T cells and western blotting with anti-GFP antibody. Retroviruses expressing Nef-EYFP or EYFP were generated by cotransfection of HEK293T cells in a T25 flask with 2 *µ*g of the transfer plasmid, 1 *µ*g of pGag-Pol, and 0.5 *µ*g of pVSVg using the calcium phosphate method. The culture supernatants were collected after 36 hr and used as the source of recombinant retroviruses. Human monocytic U937 cells were washed with RPMI, starved for 90 min without serum, and then transduced with 500 *µ*L of culture supernatants per 1 × 10^6^ cells. After 4 hrs of virus adsorption, the cells were washed and kept in complete medium for 48 hr prior to the addition of 350 ng/mL puromycin. The cells were split every 48 hr and those surviving after 5 passages were used for the analysis. The clones were sorted for the EYFP positive population using a Becton Dickinson Aria Cell Sorter in the Central Facility of the National Institute of Immunology, New Delhi, India. The sorted clones were cultured for 4-5 passages and checked for purity and YFP expression using Cyan-ADP flow cytometer (Beckman Coulter). Data was analyzed using Summit 4.3 software.

### 2.2. RNA Extraction and Microarray Hybridization

The procedural details of RNA extraction from cells and exosomes followed by the estimation of its yield and purity using spectrophotometric protocols and its RIN score using the Agilent 2100 Bioanalyzer (Agilent Technologies, Santa Clara, CA, USA) have been described elsewhere [4]. The microarray hybridization experiment was carried out at Genotypic (Bangalore, India). Total high quality RNA samples were converted to cDNA, transcribed to cRNA, labeled, and then hybridized to Agilent Whole Human Genome 8X60K microarray according to the manufacturer's recommendations. The slides were scanned according to standard protocols (Agilent). The raw data was imported into GeneSpring GX version 11.0 for further analysis. The hybridization experiment was run in triplicate for each sample.

### 2.3. Datasets and Normalization

Broadly the two datasets are cellular RNA and exosomal RNA. In the cellular mRNA dataset, there are three samples from U937 cells expressing Nef-EYFP (called mNC) and two control samples from U937 cells expressing EYFP (called mYC). The third control sample mYC was an outlier and was not considered in the analysis. The exosomal mRNA dataset has three samples for U937 cells expressing Nef-EYFP (called mNE) and three control samples from U937 cells expressing EYFP (called mYE). There are 50,238 mRNAs in each dataset. The zero-mean normalization method [16] was used to convert the genewise data from different scales to a common scale in such a way so that mean and standard deviation of the converted genewise data should be zero and one, respectively.

### 2.4. VGA and **k**-Means Clustering

Variable string length genetic algorithm (VGA) clustering [17] is a genetic algorithm-based nonparametric clustering technique that does not require specification of the number of clusters a priori. It can automatically identify the appropriate number of clusters as well as the proper partitioning of the data. Another popular clustering technique is the *k*-means algorithm [18]. It is used to evolve *k* cluster means using an iterative procedure that minimizes the sum of squared error criterion. To obtain partitioning of the data, each gene is assigned to the mean to which it is closest. Here Euclidean distance was used as a measure of the distance between two genes. As the number of samples in the mRNA data is very small while the number of genes is large, direct application of any statistical analysis on the dataset was difficult. Therefore, clustering was first applied and the resulting clusters were analyzed to identify those that represent upregulated and downregulated genes in the experimental (Nef-EYFP) samples with respect to the control (EYFP) samples for both cellular and exosomal mRNA.

### 2.5. Statistical Analysis

After using clustering to identify potential differentially expressed genes, we utilized three statistical tests [16], which included significance analysis of microarrays (SAM), *t*-test, and permuted *t*-test on the normalized data to finally identify the upregulated and downregulated genes. From a statistical perspective, the *t*-test is a standard tool for differential expression analysis when the number of samples is large. Permuted *t*-test is normally useful when there is no information on distribution of the data. For small sample sizes, application of *t*-test and permuted *t*-test may not be appropriate. Therefore, we also used SAM, a very powerful tool for small sample size of the data. Finally, we took the intersection of upregulated cellular mRNAs (referred to as mNC_up_) and the intersection of downregulated cellular mRNAs (referred to as mNC_down_) obtained from the *t*-test, permuted *t*-test, and SAM for the mNC versus mYC cellular dataset. Again, for the mNE versus mYE exosomal dataset, we followed the same procedure to obtain the upregulated mRNAs (referred to as mNE_up_) and downregulated mRNAs (referred to as mNE_down_). We refer to the nondifferentially expressed mRNAs in Nef cells and exosomes as mNC_non_ and mNE_non_, respectively.

### 2.6. Identifying Selective mRNAs

After statistical analysis, the mRNAs that are selectively secreted from Nef-expressing cells to exosomes were identified. These are referred to as mNE_sel_ while those that are selectively retained in Nef-expressing cells are referred to as mNC_sel_. The set mNE_sel_ was obtained by the intersection of mNC_down_ and mNE_up_. Similarly, the set mNC_sel_ was obtained by the intersection of mNC_up_ and mNE_down_.

### 2.7. Network Analysis

The miRNA-gene network analysis was carried out and two networks were constructed. In the first network, the genes of mNC_sel_ and the corresponding miRNAs that target them (identified using the miRWalk database) were considered. In the second network, the genes of mNE_sel_ and the corresponding miRNAs were studied. The status of the miRNA targets of significant mRNAs was verified in Nef-expressing cells/exosomes as reported in our previous study [4].

## 3. Results

### 3.1. Data Analysis

**Figure 1:**
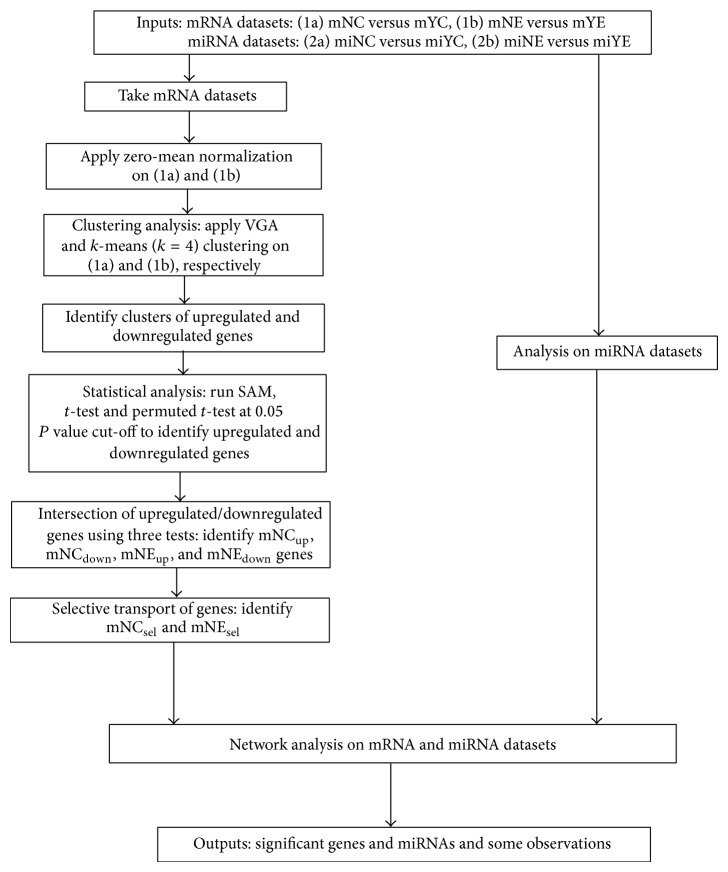
Flowchart of integrated data analysis of transcriptome and miRNome.

Data acquisition was carried out as described in Section 2. The datasets included miRNAs and mRNAs profiled from U937/Nef-EYFP cells and exosomes (test set) and from U937/EYFP cells and exosomes (control set). The mRNA analyses were then carried out as summarized in the flow chart shown in Figure 1. The miRNA analyses of exosomes secreted from Nef-expressing and control cells have been described previously. As reported earlier, we identified 47 miRNAs to be exclusively secreted in Nef exosomes and 2 miRNAs to be selectively retained in Nef-expressing U937 cells. These are considered in this study to build a bioinformatics framework towards the plausible regulation of mRNA expression through selective distribution of their targeting miRNAs in cells or exosomes.

### 3.2. Identification of mRNAs Differentially Present in Nef-Expressing Cells and Their Exosomes

**Figure 2:**
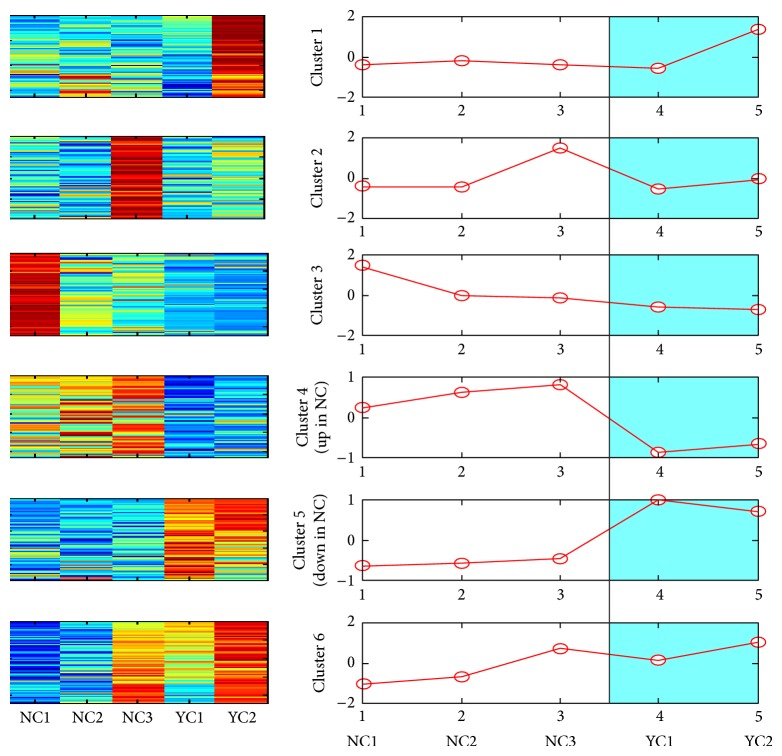
VGA clustering on mNC versus mYC, where test (Nef-expressing) cellular samples are denoted by NC1, NC2, and NC3 and control cellular samples are denoted by YC1 and YC2.

**Figure 3:**
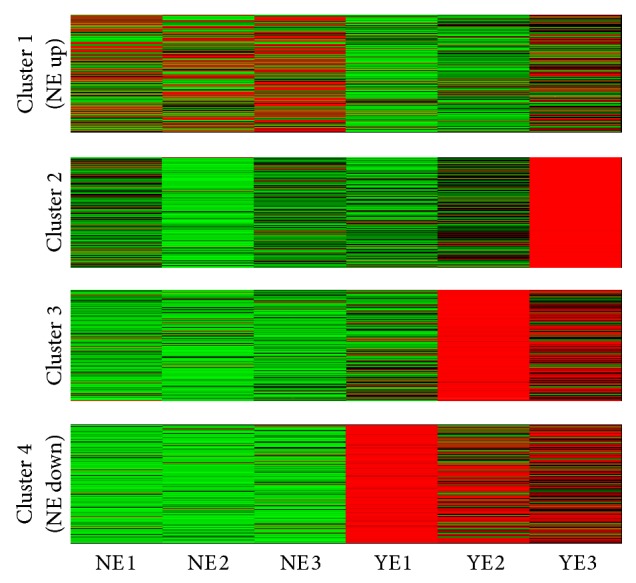
*k*-means clustering on mNE versus mYE, where test (Nef-expressing) exosomal samples are denoted by NE1, NE2, and NE3 and control exosomal samples are denoted by YE1 and YE2.

The mRNA data was normalized using zero-mean normalization followed by VGA clustering. The analysis on normalized data (mNC versus mYC) identified six clusters (Figure 2). Among these, the mRNAs in cluster 4 were upregulated (mNC_up_; *n* = 8522) and those in cluster 5 were downregulated (mNC_down_; *n* = 9816) in Nef-expressing cells. For the normalized exosome mRNA datasets (mNE versus mYE), VGA clustering was unable to identify any cluster. We therefore performed the less stringent *k*-means clustering and found four clusters on the exosomal (mNE versus mYE) dataset (Figure 3). This clearly identified mRNAs in cluster 1 to be upregulated (mNE_up_; *n* = 5385) and mRNAs in cluster 4 to be downregulated (mNE_down_; *n* = 13122) in exosomes purified from Nef-expressing cells.

**Figure 4:**
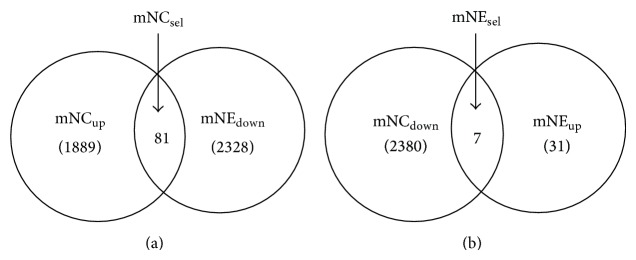
Selective transport of genes: (a) refers to the set mNC_sel_ of 81 genes and (b) refers to the set mNE_sel_ of 7 genes.

We then utilized SAM, *t*-test, and permuted *t*-test on different gene clusters in each group and determined the common genes from the three statistical tests. This provided 1889 upregulated (mNC_up_) and 2380 downregulated (mNC_down_) mRNAs in Nef-expressing cells (Table 1). Similarly, for the exosomal mRNA dataset, we recognized 31 upregulated (mNE_up_) and 2328 downregulated (mNE_down_) mRNAs in exosomes from Nef-expressing cells (Table 2). We then intersected the mNC_up_ and mNE_down_ groups (mNC_up_∩mNE_down_) to identify 81 mRNAs that are selectively retained in Nef-expressing cells and are not secreted in exosomes despite their high intracellular levels (mNC_sel_; Figure 4(a)). Similarly, we intersected the mNC_down_ and mNE_up_ groups (mNC_down_∩mNE_up_) to identify 7 mRNAs that are preferentially secreted in Nef exosomes despite their low expression levels in Nef-expressing cells (mNE_sel_; Figure 4(b)). The mRNAs belonging to the mNC_sel_ and mNE_sel_ groups are shown in Table 3.

### 3.3. Network Analysis of Selectively Expressed mRNAs and miRNAs

**Figure 5:**
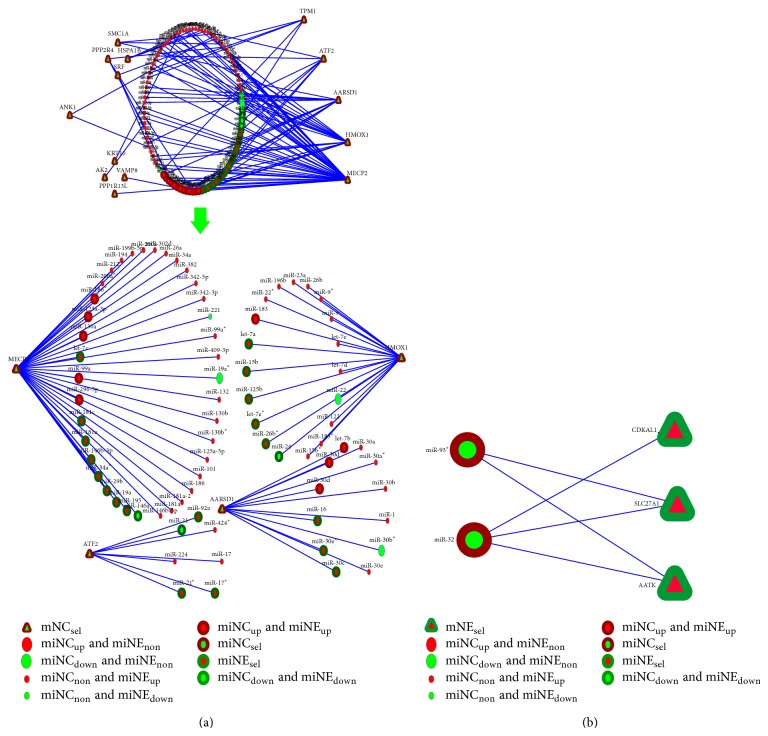
(a) There are two different views of a miRNA-target gene interaction network, where all genes are in the mNC_sel_ group and all miRNAs are those that target these genes. In the first view, the most important genes whose in-degrees (i.e., the number of miRNAs targeting the genes) are high are put on the right side of the network, and the less important genes whose in-degrees are low are put on the left side of the network. The second view of the network is a subnetwork of the above in which the top four genes and their corresponding miRNAs are presented. (b) miRNA-target gene interaction network consisting of the genes of the mNE_sel_ group and the miRNA targets.

Next, we carried out network analysis of mRNAs selectively retained in Nef-expressing cells or secreted in exosomes from these cells, and the miRNAs that target them. In the first network (Figure 5(a)), we considered the 81 mRNAs selectively retained in Nef-expressing monocytes (mNC_sel_) and the miRNAs, which target these. The top four mRNAs identified are MECP2, HMOX1, AARSD1, and ATF2 (Figure 5(a)). Among these, MECP2 (methyl CpG binding protein 2) is targeted by 38 miRNAs, of which 35 are upregulated and 8 are selectively secreted in Nef exosomes (Table 4). MECP2 encodes a nuclear protein containing methyl CpG binding domain and can specifically bind to methylated DNA [19]. It mediates transcriptional repression through interaction with histone deacetylase and the corepressor SIN3A [20]. The second most significant mRNA is HMOX1 (heme oxygenase 1) (Figure 5(a)), which is targeted by 19 miRNAs, of which 17 are upregulated and 5 are selectively secreted in Nef exosomes (Table 4). The heme oxygenase family consists of the constitutive heme oxygenase 2 and the inducible heme oxygenase 1, which is induced in response to stress stimuli [21]. The other two mRNAs produce AARSD1 (alanyl-tRNA synthetase domain-containing protein 1) and ATF2 (activating transcription factor 2) (Figure 5(a)). AARSD1 is targeted by 12 miRNAs, of which 11 are upregulated and 3 are selectively secreted in Nef exosomes (Table 4). It is a class-II aminoacyl-tRNA synthetase that functions in* trans* to edit the amino acid moiety from incorrectly charged tRNA(Ala) and thus prevents mistranslation of proteins [22]. ATF2 is targeted by 7 miRNAs, of which 6 are upregulated and 3 are selectively secreted in Nef exosomes (Table 4). The ATF/CREB family includes CREB, CRE-BP1 (ATF2), ATF3, ATF4, ATF6, and B-ATF, which are transcription factors with a common bZIP motif through which they bind to the cAMP response element (CRE) in DNA [23]. Thus the network analysis on mRNA and miRNA datasets has identified four mRNAs that are selectively retained in Nef-expressing U937 cells and the corresponding validated miRNAs that target them are selectively secreted in exosomes from these cells.

In the second network (Figure 5(b)), we considered seven mRNAs preferentially secreted in Nef exosomes (mNE_sel_) and the validated miRNAs that target their genes. We identified three mRNAs in this group, which are targeted by miRNAs that are selectively retained in Nef-expressing cells. These are AATK (apoptosis associated tyrosine kinase) and SLC27A1 (solute carrier family 27 fatty acid transporter member 1), which are targeted by miR32 and mir93^*^, and CDKAL1 (CDK5 regulatory subunit associated protein 1-like 1), which is targeted by miR32 (Figure 5(b)). The AATK is a serine/threonine kinase expressed during apoptosis [24]. It contains an N-terminal kinase domain and a C-terminal proline-rich domain, induces growth arrest and apoptosis, and can indirectly inhibit the activation of Na-K-Cl cotransporter [25]. The SLC27A1 transports long chain fatty acids across the plasma membrane, promotes the accumulation of fatty acids, and is shown to regulate cholesterol metabolism in HEK293 cells [26]. CDKAL1 is the first eukaryotic methylthiotransferase to be identified [27]. Thus, we have identified three mRNAs that are preferentially secreted in exosomes from Nef-expressing cells and find the miRNAs that target these to be upregulated within the cells.

## 4. Discussion

The HIV accessory protein Nef is a multifunctional pathogenic factor that facilitates viral replication by modulating several signalling cascades [28]. Nef is also secreted out from infected cells into exosomes [11]. Although the exact composition of the exosomal cargo depends upon the cell of origin, recent studies have shown many mRNAs and miRNAs to be highly enriched or exclusively present in exosomes [9]. We have recently published the first miRNome analysis of HIV-1 Nef-expressing human monocytic U937 cells and their exosomes [4]. We showed that Nef exosomes are enriched in miRNAs that can target proinflammatory cytokines and other genes involved in important pathways like MAPK, Jak-STAT signalling, and apoptosis. Moreover, a vast majority of miRNAs that can potentially target the HIV-1 genome were also found in exosomes secreted from Nef-expressing cells [4]. In this study, we report the first genome-wide transcriptomic analysis of Nef-expressing human monocytic cells and their exosomes. We have used clustering analysis and statistical tools to identify key mRNAs that are exclusively retained in Nef-expressing cells or are secreted into exosomes. Network analysis of these selectively enriched mRNAs and the validated miRNAs that target them was carried out to understand if gene expression is regulated by exosomal miRNA secretion. We identified four genes, namely, MECP2, HMOX1, AARSD1, and ATF2, whose mRNAs are upregulated in Nef-expressing cells and whose targeting miRNAs are preferentially secreted in exosomes from these cells. Thus, it is plausible that the expression of these genes in Nef-expressing monocytes is regulated to an extent by the secretion of their targeting miRNAs in exosomes.

A recent study showed MECP2 to interact with the transcriptional coactivator LEDGF/p75 (lens epithelium-derived growth factor p75) and influence Hsp27 promoter activation [29]. LEDGF is also a binding partner of HIV integrase and is required for correct integration of the viral genome into the host chromatin [30]. Further, methylation of the HIV genome is one of the mechanisms of viral latency [31]. It is thus tempting to speculate that Nef expression in monocytes induces MECP2 expression, which might help in maintaining the HIV genome in a latent state. Further, the MECP2 and LEDGF/P75 complex might also regulate activation of other stress survival genes like those of chaperones. The next gene identified in the network analysis was HMOX, which expresses an essential enzyme in heme catabolism. It cleaves heme to form biliverdin, which is subsequently converted to bilirubin by biliverdin reductase [32]. It plays crucial role in suppressing inflammation and protecting against oxidative stress. Upregulation of HMOX-1 is an indication of oxidative stress [33]. We have previously shown that Nef expression in monocytic cells induces the expression of proinflammatory cytokines and thus acts like a cellular stress signal. Interestingly, expression of Nef independently is capable of triggering the induction of host defense mechanisms like HMOX-1. Recently Wang et al. observed mRNAs for many tRNA synthetases and a unique splice variant in exosomes secreted from Jurkat T cells and showed that these mRNAs could be translated* in vitro* as well as in the cells that engulfed these exosomes [34]. It is now believed that a diverse pool of tRNA synthetase derived mRNAs is packaged in exosomes for genetic exchange. Besides their role in translation, tRNA synthetases also have roles in mTOR signaling, DNA repair, and apoptosis [35]. We show that the AARSD1 mRNA is preferentially retained in Nef-expressing cells, and this might regulate important signaling pathways. The next mRNA is for ATF2, which is activated by the p38 MAPK and regulates transcription of various genes involved in cell growth, response to DNA damage and survival [36, 37]. It also functions as a histone acetyltransferase and can directly interact with chromatin to regulate transcription [23]. We show that ATF2 mRNA is preferentially retained in Nef-expressing cells, and it might regulate the transcription of key genes.

We also identified the mRNAs for three proteins, AATK, SLC27A1, and CDKAL1, to be preferentially secreted out in exosomes from Nef-expressing cells and their targeting miRNAs (miR32 and miR93^*^) to be retained in these cells. Recent studies show AATK to promote apoptosis in melanoma cells and to be regulated by the Src kinase [24, 38] and in neurons to be important for recycling endosomes and synaptic vesicle transport [25, 39]. It is reported that Nef exosomes can lead to apoptosis of recipient CD4+ T cells, but no mechanism is known for this. We have also observed that these exosomes can enter different immune cells and A549 lung adenocarcinoma cells (MA and SJ; unpublished data). The selective packaging of AATK mRNA in exosomes might lead to apoptosis induction in target cells. As Nef-containing exosomes are also observed in infected individuals, this could be an important mechanism for large-scale bystander cell death of uninfected CD4 T cells observed in HIV/AIDS patients [1, 40]. It was shown recently that exosomes from HIV infected cells can activate quiescent CD4+ T lymphocytes and promote HIV-1 replication through a Nef- and ADAM17-dependent mechanism [41]. The SLC27A1 gene product is involved in the translocation of long chain fatty acids across the plasma membrane [42], which is relevant since the free fatty acid concentration is increased in HIV/AIDS patients [43, 44]. Recently, a proteomics study showed that HIV infection in T cells increases free fatty acid production and other key proteins involved in lipid metabolism [45]. Our* in silico* analysis here shows that the mRNA for fatty acid transporter protein is selectively packaged in Nef exosomes. This may lead to altered SLC27A1 production in Nef-expressing cells and can potentially increase the levels of this protein in cells receiving these exosomes. Thus, exosomal packaging of SLC27A1 mRNA could be a mechanism for regulating free fatty acid levels during HIV infection. The third mRNA is for CDKAL1, which is a member of methylthiotransferase family. While its exact function is not known, genes in this family are involved in posttranscriptional modifications of tRNA [46].

Overall, this study employs* in silico* analysis of the transcriptomic data of Nef-expressing monocytic cells and their exosomes and identifies key mRNAs that are exclusively retained in cells or secreted in exosomes. The mRNAs preferentially retained in cells are essentially involved in chromatin modification and transcriptional regulation. These pathways are critical for active gene expression as well as latent viral reservoirs. The mRNAs selectively packaged in exosomes are involved in apoptosis and fatty acid transport. These are critical for lipid metabolism and bystander cell activation and death. Thus, our* in silico* analysis has identified key intracellular and extracellular signalling pathways targeted by HIV in monocytic cells. Our study further supports the paradigm that HIV utilizes the exosomal intercellular communication network to optimize its spread in infected hosts [47, 48]. Further functional investigations are needed to validate these findings.

## 5. Conclusions

This study presents transcriptomic and network analysis of human monocytic cells expressing the HIV-1 Nef protein and their exosomes. We identified four mRNAs that are exclusively retained in Nef-expressing cells while their targeting miRNAs are exported out in exosomes. These are involved in chromatin modification, transcriptional regulation, and stress response. We also identified three mRNAs that are preferentially secreted in exosomes and whose targeting miRNAs are retained in Nef-expressing monocytes. These are involved in apoptosis induction and fatty acid transport. Monocytes are important latent viral reservoirs, and apoptosis of bystander cells and dysregulation of fatty acid metabolism are important events in HIV pathogenesis. Thus, our findings have important implications in understanding HIV pathogenesis from the triangular axis of mRNAs, miRNAs, and exosomes, which has remained poorly studied.

## Figures and Tables

**Table 1:** The number of upregulated and downregulated genes from different statistical tests in case of mNC versus mYC at 0.05 *P* value cut-off.

	SAM	*t*-test	Permuted *t*-test	Common
#mNC_up_	1891	2263	3902	1889
#mNC_down_	2667	3856	2780	2380

**Table 2:** The number of upregulated and downregulated genes from different statistical tests in case of mNE versus mYE at 0.05 *P* value cut-off.

	SAM	*t*-test	Permuted *t*-test	Common
#mNE_up_	60	179	222	31
#mNE_down_	5277	6038	3476	2328

**Table 3:** Selective transport of genes: the set mNC_sel_ of 81 genes and the set mNE_sel_ of 7 genes.

Set	# gene	Genes
mNC_sel_	81	AA418814, AARSD1, ACAD9, AK026896, AK055214, AK2, AKR1A1, ANK1, ANKRD16, ANKRD25, ANXA2, ATF2, ATP2B3, BE138567, BG114486, BLVRA, C1orf131, C6orf106, C9orf23, CDC5L, COMMD4, CRIP1, DYNC1H1, EHD2, ENST00000292140, GOLGB1, GSTO1, HELZ, HMOX1, HN1, HPS4, HSP90AB1, HSPA1B, HsapiAL110166, HsapiCR624471, KCTD1, KRT10, LMNA, LOC148413, LOC389493, LYRM4, MALAT1, MDP-1, MECP2, MPI, MRPL39, NDE1, NDUFA8, NT5M, OBSCN, OSBPL1A, PDCD7, PFDN1, PLA2G4B, POLD4, PPP1R13L, PPP2R4, QPRT, RAB1A, RACGAP1, RAD21, RBM17, RBM18, RDM1, RMRP, RPL10, SETDB2, SIGLECP3, SMC1A, SNAPC3, SRF, STAU2, SYF2, THC2541678, TK1, TMCO3, TNNT1, TPM1, UPF3B, VAMP8, ZYG11B

mNE_sel_	7	AATK, CDKAL1, GGTLA1, HsapiAJ412045, LOC196463, MTMR11, SLC27A1

**Table 4:** Some of the most important genes in mNC_sel_ and the corresponding miRNAs (which target those genes) and their status in Nef-EYFP-expressing cells and exosomes.

Gene	# miRNAs which target the gene	miRNAs which target the gene	Status of miRNAs in Nef-EYFP-expressing cells and exosomes
MECP2 (mNC_sel_)	8	*miR-29b, miR-146b-3p, let-7c, miR-19a, miR-195, miR-181c, miR-181a, miR-34a* ^*^	miNE_sel_
	22	miR-130b^*^, miR-302d, miR-382, miR-342-3p, miR-146b-5p, miR-181a^*^, miR-26a, miR-125a-5p, miR-409-3p, miR-130b, miR-199b-5p, miR-181a-2^*^, miR-186, miR-194, miR-200a^*^, miR-200a, miR-101, miR-212, miR-342-5p, miR-34a, miR-99a^*^, miR-132	miNC_non_ and miNE_up_
	1	miR-221	miNC_non_ and miNE_down_
	1	miR-19a^*^	miNC_down_ and miNE_non_
	5	miR-186^*^, miR-99a, miR-125a-3p, miR-130a, miR-296-5p	miNC_up_ and miNE_up_
	1	miR-146a	miNC_down_ and miNE_down_

HMOX1 (mNC_sel_)	5	*miR-26b* ^*^ *, miR-15b, let-7e* ^*^ *, miR-125b, let-7a *	*miNEsel *
	11	miR-122, miR-15b^*^, miR-9, miR-196b, let-7e, miR-22^*^, miR-9^*^, miR-23a, miR-183^*^, let-7d, miR-26b	miNC_non_ and miNE_up_
	1	miR-22	miNC_down_ and miNE_non_
	1	miR-183	miNC_up_ and miNE_up_
	1	miR-24	miNC_down_ and miNE_down_

AARSD1 (mNC_sel_)	3	*miR-16, miR-30e* ^*^ *, miR-30c *	miNE_sel_
	5	miR-30b, miR-30a, miR-30a^*^, miR-1, miR-30e	miNC_non_ and miNE_up_
	1	miR-30b^*^	miNC_down_ and miNE_non_
	3	miR-30d^*^, miR-30d, let-7b	miNC_up_ and miNE_up_

ATF2 (mNC_sel_)	3	*miR-17* ^*^ *, miR-92a, miR-21* ^*^	miNE_sel_
	3	miR-17, miR-224, miR-424^*^	miNC_non_ and miNE_up_
	1	miR-21	miNC_down_ and miNE_down_
